# Supplementary material for: Probing a label-free local bend in DNA by single molecule tethered particle motion
Source: Nucleic Acids Res. 2015 Mar 12;43(11):e72. doi: 10.1093/nar/gkv201 (PMC4477641; doi:10.1093/nar/gkv201)
Supplement: SUPPLEMENTARY DATA [file supp_43_11_e72__index.html]

Probing a label-free local bend in DNA by single molecule tethered particle motion — Probing a label-free local bend in DNA by single molecule tethered particle motion — SUPPLEMENTARY DATA 

# Probing a label-free local bend in DNA by single molecule tethered particle motion

## SUPPLEMENTARY DATA

**Files in this Data Supplement:**

- SUPPLEMENTARY DATA
- SUPPLEMENTARY DATA
