# Supplementary material for: Probing a label-free local bend in DNA by single molecule tethered particle motion
Source: Nucleic Acids Res. 2015 Mar 12;43(11):e72. doi: 10.1093/nar/gkv201 (PMC4477641; doi:10.1093/nar/gkv201)

SUPPORTING INFORMATION

SI text

List of abbreviations

$\vec{R}_{exp\|raw}$: 2D-vector positions of the particle measured experimentally

$\boldsymbol{R_{exp\|\mathrm{raw}}}$ Amplitude of motion of the particle defined as $\sqrt{\langle \vec{R}^2_{exp\|raw} \rangle}$

$\boldsymbol{R_{exp\|}}$ Amplitude of motion of the particle corrected from the blurring effect

$\boldsymbol{\vec{R}_{sim\|}}$ 2D-vector positions of the simulated particle

$\boldsymbol{R_{sim\|}}$ Amplitude of motion of the simulated particle defined as $\sqrt{\langle \vec{R}^2_{sim\|} \rangle}$

$\boldsymbol{R_{DNA}}$ End-to-end distance of the DNA molecule

$\boldsymbol{R_P}$ Radius of the labelling particle

$\boldsymbol{L}$ Contour length of the DNA molecule

$\boldsymbol{l}$ Distance separating the kink to one extremity of the DNA molecule

$\boldsymbol{\theta}$ Bend angle

$\boldsymbol{L_P}$ Persistence length of the DNA molecule

DNA constructs

The sequences of the 88bp long insert (Fig. 1) are the following ones:

6An0 : GCTTCCATCTCGAACCGACGTTGCTGGCCGTACATGTGGCTAGCACCATGGAGGATCCCTTAACGCTATGGAACTCGCCGCCCGACTGGGCTGTCGACCG

6An1^P: GCTTCCATCTCGAACCGACGTTGCTGGCCGTACATGTGGCTAG**CAAAAAACGG**GATCCCTTAACGCTATGGAACTCGCCGCCCGACTGGGCTGTCGACCG

6An2^P: GCTTCCATCTCGAACCGACGTTGCTGGCCGTAGGCTAG**CAAAAAACGGCAAAAAACGG**GATCCCGCTATGGAACTCGCCGCCCGACTGGGCTGTCGACCG

6An3^P: GCTTCCATCTCGAACCGACGTTGCTGGCGCTAG**CAAAAAACGGCAAAAAACGG**G**CAAAAAACGG**GATCCGGAACTCGCCGCCCGACTGGGCTGTCGACCG

6An4^P: GCTTCCATCTCGAACCGACGTTGGCTAG**CAAAAAACGGCAAAAAACGG**G**CAAAAAACGGCAAAAAACGG**GATCCTCGCCGCCCGACTGGGCTGTCGACCG

6An6^P: GCTTCCGACGTTGGCTAG**CAAAAAACGGCAAAAAACGG**G**CAAAAAACGGCAAAAAACGG**G**CAAAAAACGGCAAAAAACGG**GATCCTCGCCGCGTCGACCG

6An7^P: AAGCTTTTGGCTAG**CAAAAAACGGCAAAAAACGG**G**CAAAAAACGGCAAAAAACGG**G**CAAAAAACGGCAAAAAACGG**G**CAAAAAACGG**GATCCTCGTCGAC

6An4^O: GCTTCCATCTCGAACCGCTAG**CAAAAAACGG**ACGTA**CAAAAAACGG**ACGTA**CAAAAAACGG**ACGTA**CAAAAAACGG**GATCCCCGACTGGGCTGTCGACCG

Assembly of the HT-TPM biochip

Patterning of anchoring sites on functionalized coverslips

Regular arrays of rhodamine-labelled neutravidin (Molecular Probes) were obtained using a standard micro-contact printing protocol. Briefly, a stamp made of PDMS with squared pillars of 0.8 µm size and 3 µm pitch was inked with a 20 µg/mL neutravidin solution in PBS (Euromedex) for 1 min, then washed with deionized water and dried under nitrogen flow. The stamp was then brought into close contact with epoxidized glass support for 1 min during which the protein is transfered onto the surface.

Formation of DNA-bead complexes

Polystyrene carboxylated beads (Merck) of (150 ± 3) nm radius were covalently coated with anti-digoxigenin antibodies (Roche) using EDAC (Sigma-Aldrich), activation and storage buffers (Ademtech). A 100 pM solution of suspended functionalized beads was mixed with an equal volume of a solution of 50 pM DNA bearing a digoxigenin on one end for 20 min at room temperature in PBS buffer supplemented with 1mg/mL of pluronic 127 (Sigma-Aldrich) and 0.1 mg/mL BSA (Sigma-Aldrich), noted TPM buffer. This lead to pre-assembled DNA-bead complexes.

Assembly of the fluidic observation chamber for HT-TPM experiments

A 250 µm thick silicone tape was cut and used as a spacer between the patterned coverslip and an epoxidized glass slide with 2 holes for inlet and outlet to obtain a working flow cell. The so-formed analysis chamber was rinsed and incubated with TPM buffer for 30 min at room temperature. The DNA-bead complexes solution was introduced in the flow cell and incubated over night at 4°C.

Prior to visualization, the flow cell was extensively rinsed by injecting 30 chamber volumes of TPM buffer. Then, for each condition, movies of 5 min were recorded on different zones in the same flow cell and analyzed. To ensure reproducibility, experiments were repeated on different days.

HT-TPM experimental setup

Instrumentation for microscopy imaging

The tethered beads were visualized using a dark-field microscope (Axiovert 200, Zeiss) equipped with a x32 objective and an additional x1.6 magnification lens and acquired for 5 min at room temperature, at a recording rate of 25 Hz and with a duration of acquisition of 40ms, on a CMOS camera Dalsa Falcon 1.4M100. The field of observation covers an area of ~ 215 µm x 160 µm.

Single particle tracking

The software Nanomultiplex co-developed with Magellium Toulouse (request should be addressed at info@magellium.fr) tracks in real time the positions of all the particles using the centroid method, averages these absolute positions on a 5 s window giving access to the anchoring point of the DNA molecule, calculates the 2D-vector positions of the bead $\vec{R}_{exp\|raw}$ relative to the anchoring point of the DNA which corrects for experimental drift, calculates the asymmetry factor of the bead trajectories (S) (Blumberg S, Gajraj A, Pennington MW, Meiners J-C (2005) Three-Dimensional Characterization of Tethered Microspheres by Total Internal Reflection Fluorescence Microscopy. Biophys J 89(2):1272–1281), and the amplitude of motion of the particle defined as $\sqrt{\langle \vec{R}^2_{exp\|raw} \rangle}$ and noted $R_{exp//raw}$. In a general manner, we will use indifferently $\sqrt{\langle \vec{R}^2 \rangle}$ and R in the following.

The averages, performed in the calculation of the asymmetry factors and amplitudes of motion, are taken over a sliding window of 5 s along the time trace. We invite the reader to refer to (Plenat T, Tardin C, Rousseau P, Salome L (2012) High-throughput single-molecule analysis of DNA-protein interactions by tethered particle motion. Nucleic Acids Research 40(12):e89–e89) for the detailed calculations of $R_{exp//raw}$ of the bead.

Procedure of analysis for HT-TPM experimental data

In order to quantify the small differences expected on $R_{exp//raw}$, we set up a two-step procedure that is described in detail below. Briefly, it consists in selecting traces fulfilling several criteria of validity

and applying corrections for detector temporal averaging to their $R_{exp//raw}$. All this procedure was performed with homebuilt Mathematica scripts (available upon request).

Criteria of validity of the DNA-bead complexes

First, we discard the trajectories that have mean asymmetry factors above 1.35, calculated as the average of the asymmetric factors measured along the time trace, or that have a mean amplitude of motion smaller than 1 nm or higher than 1000 nm. Then the probability distribution of the average of for each trajectory is built with the remaining trajectories and fitted by a Gaussian distribution centered on a mean value, called mean with a standard deviation (sd). As we noticed that a few trajectories had $R_{exp//raw}$ averages standing out of the Gaussian distribution, we added a second step of validation to eliminate the misformed tethers with an average $R_{exp//raw}$ outside the interval (mean ± 2.5 sd). Using this criterion, no more than 1.3% of valid trajectories were eliminated during this additional step.

In total, about 12% trajectories were eliminated and the final number of valid trajectories eventually ranged between 348 and 3496 (See Table 1), depending on the DNA construct.

Correction of time averaging effect

Finite exposure time of detectors, T_{ex}, equal here to 40 ms, can lead to a blurring effect in single molecule (or particle) tracking experiments, as investigated for example in (Manghi M, et al. (2010) Probing DNA conformational changes with high temporal resolution by tethered particle motion. *Physical Biology* 7(4):046003). The correlation time of the positions, of about 20 ms, was calculated for each DNA-particle complex and then injected in Eq. 1 to correct the amplitudes of motion recorded on each trace.

$$R_{exp\parallel} = R_{exp\parallel raw}\left[2\frac{\tau_{\parallel}}{T_{ex}} - 2\left(\frac{\tau_{\parallel}}{T_{ex}}\right)^2\left(1 - e^{-\frac{T_{ex}}{\tau_{\parallel}}}\right)\right]^{-1/2} \quad (1)$$

As previously, the trajectories with an average $R_{exp\parallel}$outside the interval (mean ± 2.5 sd) were eliminated.

Calculation of the amplitude of the motion of an ensemble of particles

Lastly, the experimental value of the amplitude of the motion of an ensemble of particles was obtained by fitting the probability distribution of $R_{exp\parallel}$ with a Gaussian, which gave us its mean value as its center. The error on the amplitude of the motion of an ensemble of particles was calculated by using the bootstrap method of R software (R Foundation for Statistical Computing, Vienna, Austria). Doing so, we find a typical error of 0.4 nm (See Table 1).

TPM simulations

DNA coarse-grained model

We performed Kinetic Monte Carlo simulations (Newman MEJ, Barkema GT (1999) Monte Carlo Methods in Statistical Physics (Oxford University Press)) on the particle-DNA complex to predict the particle to anchor 2D-distance. The labeled DNA polymer is modeled as a chain of *N* connected small spheres of radius a, with a DNA contour length equal to $L = 2a(N-1)$, and a larger particle of radius $R_p =$ 150 nm $\geq a$ at its terminus. At this level of modeling, the internal structure of the double-stranded DNA is not considered and the persistence length, L_p, is averaged over the nucleotide sequence and taken equal to 147 bp. To model the angle imposed in the experiments by the *n* successive 10-bp CA_6CGG inserts, we have incorporated a bend of fixed angle θ between the three small beads located in the middle of the DNA molecule, which is simulated using a coarse-grained model. More precisely, the elastic term between triplets $(i-1, i, i+1)$ of successive beads, making an angle θ_i, namely $U_i = \kappa\,(1-\cos\theta_i)$, is replaced by $U_i' = \kappa\,(1-\cos(\theta_i - \theta))$ for the central triplet where κ is the bending modulus and $\kappa = 147 k_B T$. The polymer is grafted on a surface. Since the polymer motion is limited to the upper half-plane, we impose a “hard wall” boundary condition for monomer spheres and for the particle. All spheres interact via stretching and bending forces. We invite the reader to refer to (Manghi M, et al. (2010) Probing DNA conformational changes with high temporal resolution by tethered particle motion. Physical Biology 7(4):046003) for the details of these Kinetic Monte Carlo simulations.

Simulated bent DNA

The bent sequences used in the experiments are simulated by setting a fixed angle between three successive monomers located at the center of the DNA molecule. A full range of angles were studied in successive simulations: 0, 18, 30, 45, 50, 60, 72, 90, 120 and 180 degrees.

Extraction/Calculation of the particle to anchor 2D-distance of the simulated particle

The 2D-vector of the particle position $\vec{R}_{sim\parallel}$ is measured throughout simulations and utilized to estimate the amplitude of motion defined as $\sqrt{\langle \vec{R}_{sim\parallel}^2 \rangle}$, the average being taken along the trajectory.

Theory for a local bend

We consider a homogeneous polymer of length L using the Worm-Like Chain (WLC) model by Kratky and Porod with a persistence length Lp (Kratky O, Porod G (1949) Röntgenuntersuchung gelöster Fadenmoleküle. Recl Trav Chim Pays-Bas 68(12):1106–1122). We suppose that a kink is located at distance *l* from one end and locally induces a spontaneous curvature with an angle θ, see below.

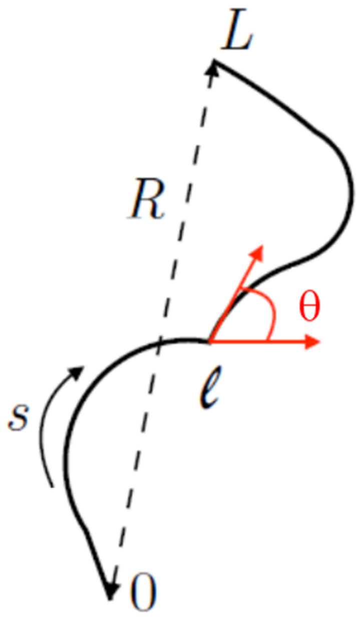

We denote the tangent vector at curvilinear position s by $\overrightarrow{\mathrm{t(s)}}$, and the tangent-tangent correlation function is given by $\langle \overrightarrow{\mathrm{t(s)}}.\overrightarrow{\mathrm{t(s')}} \rangle = exp[-|s-s'|/L_P]$.

The mean-square end-to-end distance is defined as : $\langle R^2 \rangle = \int_0^L ds \int_0^L ds' \langle \overrightarrow{\mathrm{t(s)}}.\overrightarrow{\mathrm{t(s')}} \rangle$.

Integration leads to the following result:

$$\langle R^2 \rangle = 2\, L_P^2 \left[\frac{L}{L_P} - 2 + e^{-\frac{l}{L_P}} + e^{-\frac{L-l}{L_P}} + cos(\theta)\left(1 - e^{-\frac{L-l}{L_P}} - e^{-\frac{l}{L_P}} + e^{-\frac{L}{L_P}}\right)\right]$$

Theory for a local stiffer insert

We use the same model and assume now that a stiffer insert of length b and persistence length L_{Pb} is inserted at position l, see below:

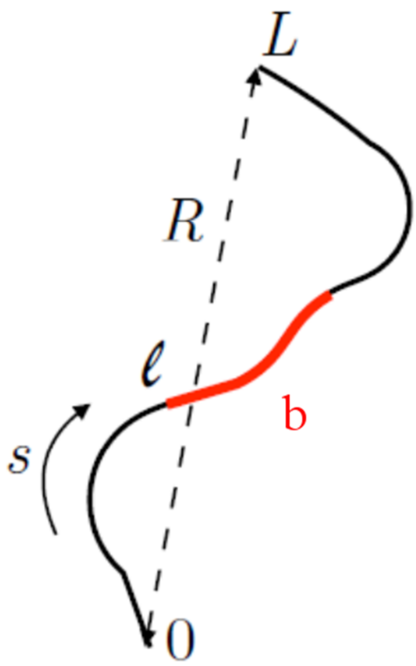

The mean-square end-to-end distance is again $\langle R^2 \rangle = \int_0^L ds \int_0^L ds' \langle \overrightarrow{t(s)}.\overrightarrow{t(s')} \rangle$. To take into account the 3 parts of the DNA molecule, the calculation requires the equation to be cut into parts. After a straightforward integration, one gets:

$$\langle R^2 \rangle = 2\, L_P^2 \left[\frac{L-b}{L_P} + e^{-\frac{l}{L_P}} + e^{-\frac{L-b-l}{L_P}} - 2 + e^{-\frac{b}{L_{Pb}}} \left(1 - e^{-\frac{l}{L_P}}\right)\left(1 - e^{-\frac{L-b-l}{L_P}}\right) + 2 L_P L_{Pb} \left(1 - e^{-\frac{b}{L_{Pb}}}\right)\left(2 - e^{-\frac{l}{L_P}} - e^{-\frac{L-b-l}{L_P}}\right)\right] + \ 2 L_{Pb}^2 \left(\frac{b}{L_{Pb}} - 1 + e^{-\frac{b}{L_{Pb}}}\right)$$

S1 figure: linearity of R_{DNA} with the length of a rigid insert. Simulated data are represented by black markers and the linear fits by red straight lines**.**

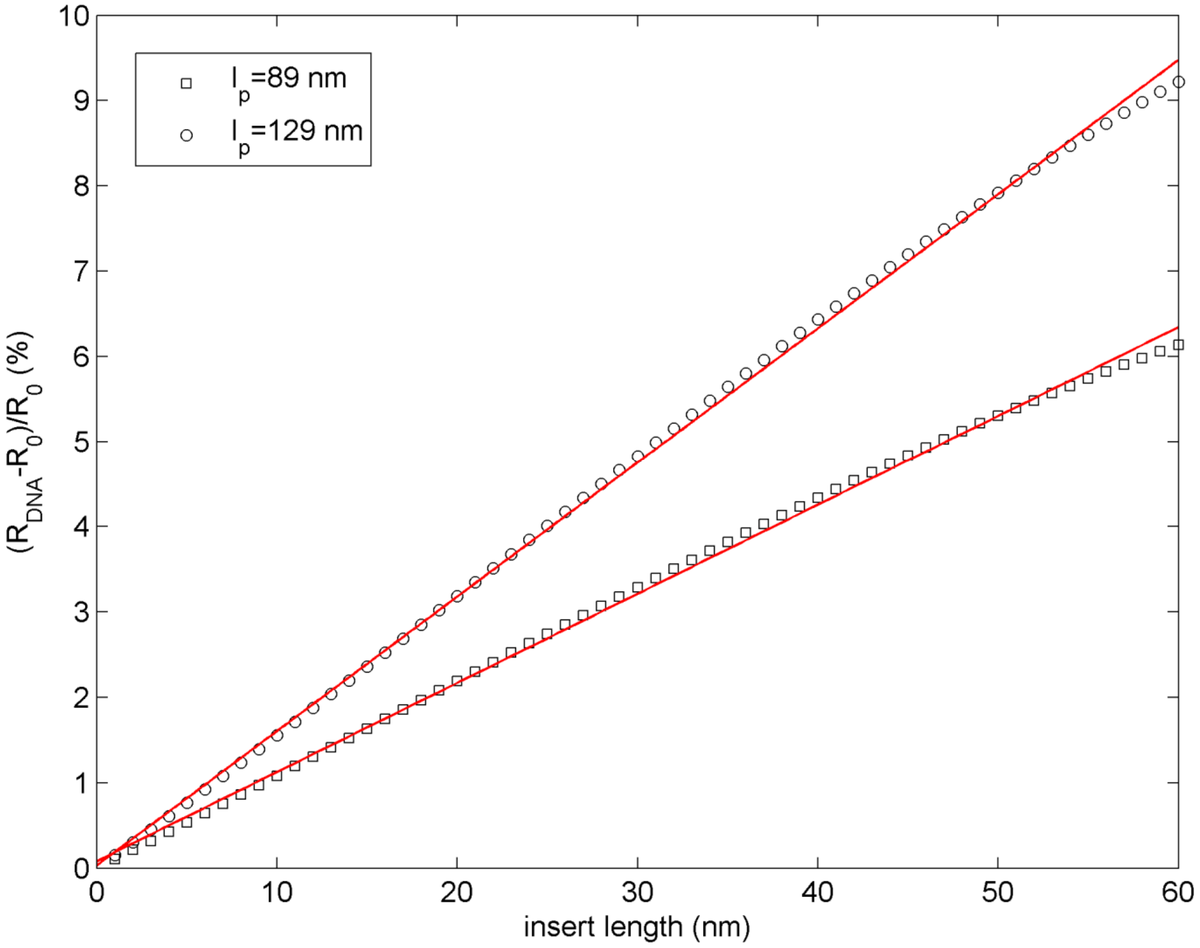

S2 figure: complement to Fig. 3

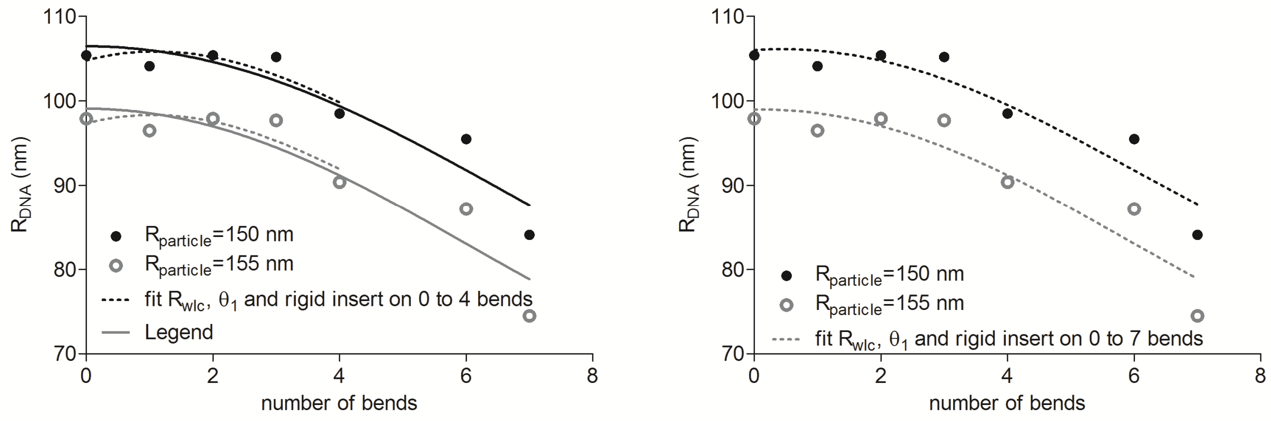

Experimental data (symbols) obtained after deconvolution of the particle effects using the minimal model and considering the size of labelling particle equal to 150 nm (●) and 155 nm (○), and two series of fits with R_{WLC} and θ_1 (–) (already shown in figure 3), or R_{WLC}, θ_1 and a rigidity term α (…) as free parameters. The results are shown in S3 Table.

S3 table: complement to S2

$$\sqrt{\langle R_{WLC}^2 \rangle} = D[(1 + 0.338\, cos(n\theta_1 - \theta_0)\,]^{1/2} + \, \alpha n$$

**Fit conditions**	R_p	D (nm)	θ_1 (°)	θ_0 (°)	α (nm)
No global curvature ($\theta_0 = 0$)<br>No local rigidity ($\alpha = 0$)	150	92 ± 2	15 ± 2		
	155	86 ± 2	17 ± 2		
No local rigidity ($\alpha = 0$)	150	91 ± 2	19 ± 4	25 ± 19	
	155	95 ± 2	21 ± 4	29 ± 22	
No global curvature ($\theta_0 = 0$)<br>Data fitted from n =0 to 4	150	91 ± 2	17 ± 12		2 ± 4
	155	84 ± 3	22 ± 15		2 ± 4
No global curvature ($\theta_0 = 0$)<br>Data fitted from n =0 to 7	150	92 ± 3	17 ± 12		1 ± 4
	155	86 ± 3	17 ± 15		0 ± 4

S4 figure: non-linearity of R_{DNA} with the persistence length of a rigid insert.

R_{DNA} is computed considering: L=575 bp , l=274bp, b=88bp and with L_p=150 bp outside the 88bp insert. As a result, R_{DNA} appears to be poorly sensitive to an increase of rigidity of the insert for persistence lengths of the insert > 150 bp.

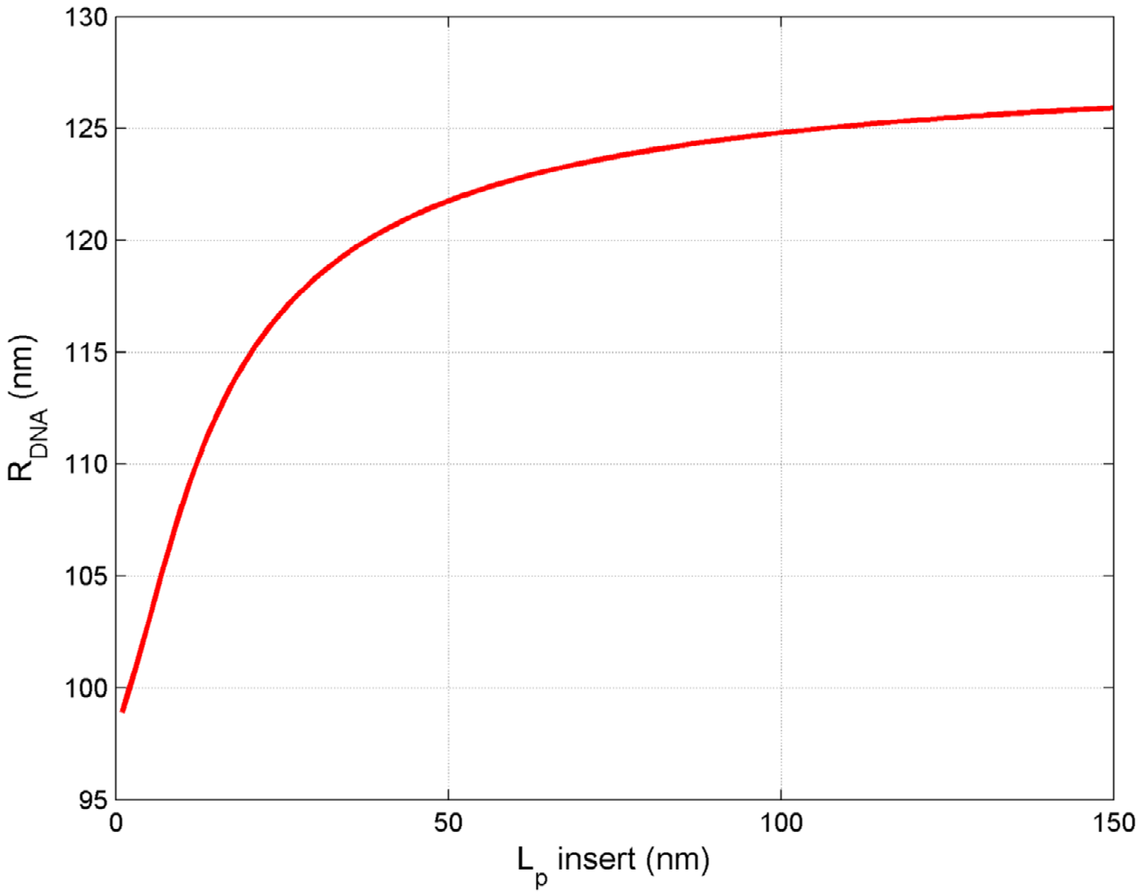

S5 table

DNA test	DNA reference	$R_{DNAtest}$	R_{DNAref}	$\frac{\left[\langle R^2_{DNA}\rangle_{REF} - \langle R^2_{DNA}\rangle_{TEST}\right]}{\langle R^2_{DNA}\rangle_{REF}}$	θ (°)	$\frac{\theta\ (°)}{n}$
$6An4^P$	6An0	98.5	105.4	0.127	60	15
$6An6^P$	6An0	95.5	105.4	0.179	73	12
$6An7^P$	6An0	84.1	105.4	0.363	116	17
$6An4^O$	6An0	108.6	105.4	-0.062	-	-

Video S1

Typical field of Particle-DNA complexes recorded by HT-TPM (area size about 100 µm X150 µm)

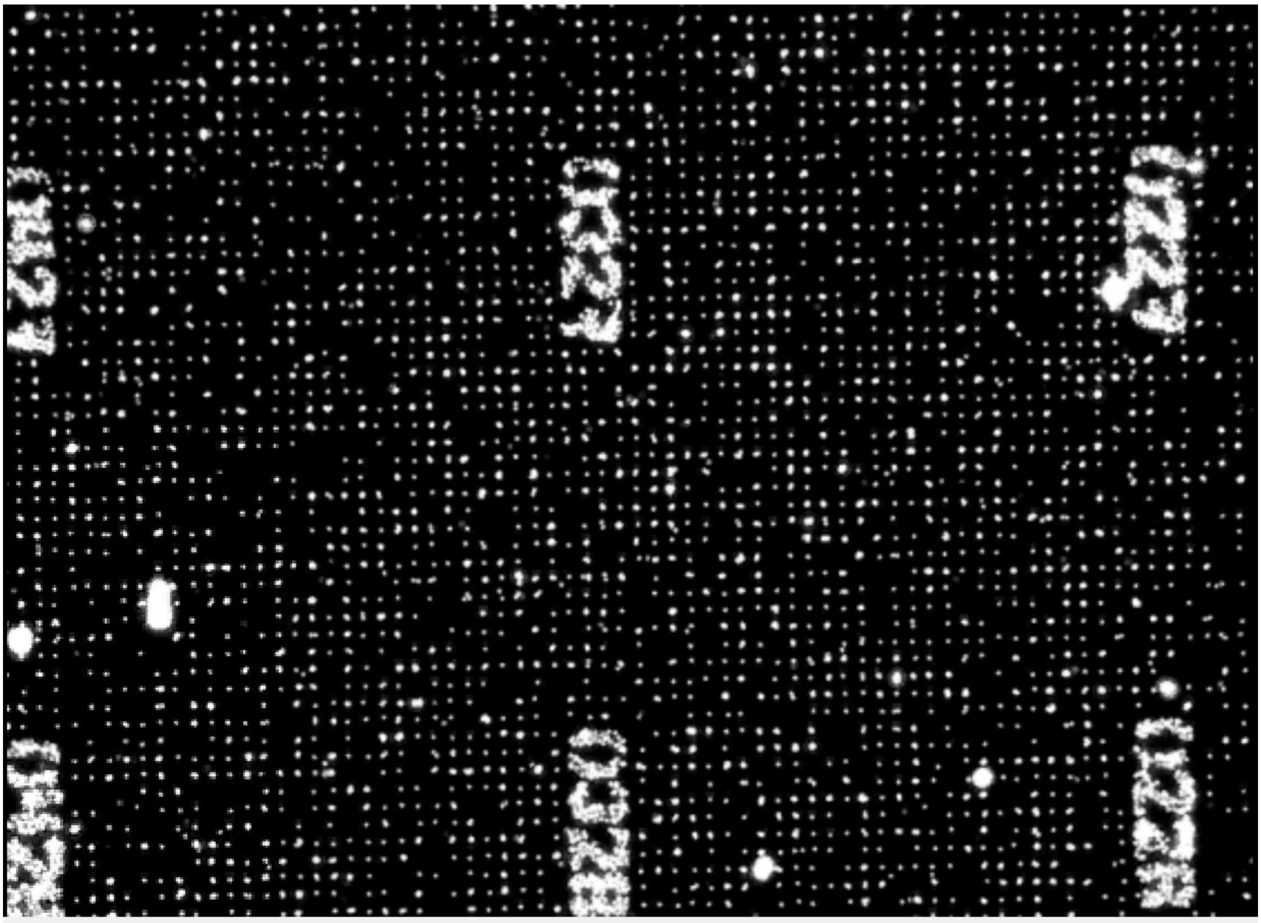

Supplement: SUPPLEMENTARY DATA [file supp_gkv201_nar-00025-met-f-2015-File005.pdf]
